# Relation between Speech-in-Noise Threshold, Hearing Loss and Cognition from 40–69 Years of Age

**DOI:** 10.1371/journal.pone.0107720

**Published:** 2014-09-17

**Authors:** David R. Moore, Mark Edmondson-Jones, Piers Dawes, Heather Fortnum, Abby McCormack, Robert H. Pierzycki, Kevin J. Munro

**Affiliations:** 1 NIHR Nottingham Hearing Biomedical Research Unit, Nottingham, United Kingdom; 2 MRC Institute of Hearing Research, University Park, Nottingham, United Kingdom; 3 Cincinnati Children's Hospital Medical Center and Department of Otolaryngology, University of Cincinnati College of Medicine, Cincinnati, Ohio, United States of America; 4 Otology and Hearing Group, Division of Clinical Neuroscience, School of Medicine, University of Nottingham, Nottingham, United Kingdom; 5 School of Psychological Sciences, University of Manchester, Manchester, United Kingdom; 6 Central Manchester University Hospitals NHS Foundation Trust, Manchester Academic Health Science Centre, Manchester, United Kingdom; UNLV, United States of America

## Abstract

**Background:**

Healthy hearing depends on sensitive ears and adequate brain processing. Essential aspects of both hearing and cognition decline with advancing age, but it is largely unknown how one influences the other. The current standard measure of hearing, the pure-tone audiogram is not very cognitively demanding and does not predict well the most important yet challenging use of hearing, listening to speech in noisy environments. We analysed data from UK Biobank that asked 40–69 year olds about their hearing, and assessed their ability on tests of speech-in-noise hearing and cognition.

**Methods and Findings:**

About half a million volunteers were recruited through NHS registers. Respondents completed ‘whole-body’ testing in purpose-designed, community-based test centres across the UK. Objective hearing (spoken digit recognition in noise) and cognitive (reasoning, memory, processing speed) data were analysed using logistic and multiple regression methods. Speech hearing in noise declined exponentially with age for both sexes from about 50 years, differing from previous audiogram data that showed a more linear decline from <40 years for men, and consistently less hearing loss for women. The decline in speech-in-noise hearing was especially dramatic among those with lower cognitive scores. Decreasing cognitive ability and increasing age were both independently associated with decreasing ability to hear speech-in-noise (0.70 and 0.89 dB, respectively) among the population studied. Men subjectively reported up to 60% higher rates of difficulty hearing than women. Workplace noise history associated with difficulty in both subjective hearing and objective speech hearing in noise. Leisure noise history was associated with subjective, but not with objective difficulty hearing.

**Conclusions:**

Older people have declining cognitive processing ability associated with reduced ability to hear speech in noise, measured by recognition of recorded spoken digits. Subjective reports of hearing difficulty generally show a higher prevalence than objective measures, suggesting that current objective methods could be extended further.

## Introduction

The detection of quiet tones of varying frequency, has for >70 years been the gold standard test of hearing [1]. However, the most frequent complaint expressed by people about their hearing is inability to follow speech in noisy environments [2]. Pure-tone audiograms, measures of tone detection threshold across frequency [2], and speech perception measured in the quiet [3], do not predict well the handicap produced by hearing loss. These principles were first recognized long ago [4]–[7], as was the finding that speech-in-noise (SiN) hearing ability decreases with age [3], even while the audiogram may remain relatively stable [4]. SiN measures that use familiar speech correlate with the average level of hearing based on the pure-tone audiogram (the ‘pure tone average’, PTA [8]–[10]) and may offer an alternative gold standard more relevant to everyday hearing. Despite much research, SiN hearing has until recently received little attention in large population studies and limited clinical application. However, the development of the Digit Triplets Test (DTT [11]), a measure of SiN hearing that can be administered without specialist supervision and in any quiet room (go to actiononhearingloss.org.uk for a demonstration and test), enabled inclusion in UK Biobank [12], [13]. UK Biobank is an internationally accessible data resource based on a very large-scale, ongoing, longitudinal study across England, Scotland and Wales of many aspects of health, starting in middle age (40–69 year olds; www.ukbiobank.ac.uk).

We report here on the baseline UK Biobank DTT data, compared in the same participants with other UK Biobank data on cognitive ability and self-reported hearing, and with other large, audiogram-based studies [14]–[18]. Paraphrasing Neisser [19], cognition is the transformation of sensory information into meaning. It may refer to a broad range of constructs relevant to hearing including attention, memory, intelligence, learning, processing speed and language. Language skills were not tested directly in UK Biobank, but they undoubtedly contributed to some extent to the self-report questions on hearing, the DTT, and all the cognitive tests, especially some items in the test of Fluid Intelligence (Fig. S3 in Results S1).

We first asked whether the increase in tone threshold with age, previously measured by the audiogram and by sentences in noise [20], is paralleled by an increase in DTT speech reception threshold (DTT SRT). Given the presumed greater cognitive demand of DTT, involving speech recognition and working memory, we predicted that an increase in DTT SRT with age would be greater than the relative increase in PTA because of a combination of reduced audibility and reduced cognition [21]. This prediction bears on a fundamental clinical issue, how best to measure and treat hearing impairment. This is a timely issue because of the increase of hearing-related problems in an aging population [21] and the large proportion of middle age people with significant and treatable hearing loss who currently remain untreated [22].

**Figure 1:**
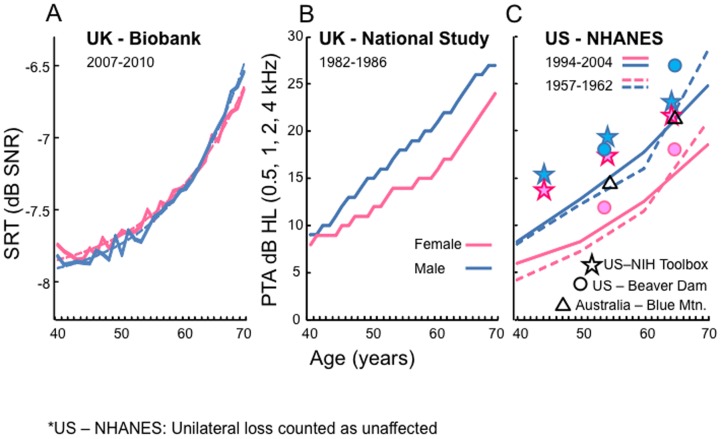
Hearing declines from 40–69 years of age. (A) UK Biobank: Mean DTT speech reception threshold (SRT; better ear) data, corrected for differences in socio-economic between samples. Exponential functions with an additive constant are fitted to the data. (B) National Study of Hearing (UK [14]): Mean pure tone average (PTA) thresholds (0.5, 1, 2, 4 kHz; better ear). (C) National Health and Nutrition Examination Survey (NHANES, US [16]): Mean PTA thresholds (0.5–4 kHz). Other data points from NIH Toolbox (US, 2011 [18]), Beaver Dam Epidemiology of Hearing Loss Study (US, 1993 [15]), Blue Mountains Hearing Study (Australia, 1997–2000 [17]).

Sensitivity to high frequency tones begins to decline in most people by 30–40 years old [16], [18] then spreads to lower frequencies from 40 years onwards (Fig. 1B, C). Overall, 21% of UK 40–69 year olds had clinical hearing loss (PTA>20 dB hearing level, HL, 0.5–4.0 kHz in better ear) in the mid-1980s [14], the last time it was measured in a large UK population. The version of the DTT used in UK Biobank, like the original telephone test, has a high frequency limit of 4.0 kHz, so is a suitable test for comparison with those historical data. Cognitive function is most commonly measured using IQ, processing speed and working memory tests [23]. In UK Biobank, cognition is assessed using visually delivered measures of fluid intelligence, processing speed, executive function, number storage (digit span), and visuospatial working memory (shape pairs matching). It is not currently known which aspects of cognition are most closely related to hearing but the broad cognitive categories tested in UK Biobank have all been implicated [24], [25]. We report here the relationships between those categories and DTT SRT in middle age.

SiN hearing varies significantly in difficulty, and everyday relevance, with the nature of both the speech (typically syllables, words or sentences) and the noise (e.g. ‘white’, speech-shaped, modulated, or real, competing speakers). The DTT choice of single digit words promotes easy understanding and the opportunity to ‘fill-in’ missed acoustic information, while steady-state, speech-shaped noise enables masking across the speech range, but does not allow the ‘glimpsing’ that can reduce masking in more complex noise. Overall, DTT performance correlates more closely with PTA than do some other SiN tests [10]. The DTT is reproducible, easy to standardize, available in several languages, does not require a sound booth, and may be delivered through the internet. However, it has been suggested [9], [10] that it may not be as sensitive to cognitive function as some other SiN tests.

Self-report of hearing difficulties, using questionnaire instruments or often only one or two questions, has commonly been used to assess the prevalence of hearing loss in large epidemiological studies [14], [26]. Some studies [14], [27] have reported the relationship of self-report with audiometric measures but only rarely have self-report data been validated against standard audiometric measures [28]–[30]. Self-report assessed by a single question (‘Do you feel you have a hearing loss?’) has been reported to demonstrate high sensitivity and specificity. A multi-question hearing handicap inventory had lower sensitivity but higher specificity and a positive predictive value. Both question and inventory were recommended for assessing burden but could not measure hearing loss [28].

In this study we investigated the relation between DTT SRT and cognitive ability as a function of age, sex, socioeconomic status, and noise exposure. The results were compared with self-report of hearing difficulties and findings from other studies, including some previously unpublished data from the NIH Toolbox [18], [23], of age-related changes in tone sensitivity and cognitive performance. It was predicted that DTT SRT would decline with age more rapidly than tone sensitivity and that the decline in DTT SRT would be more closely associated with increased self-reported hearing difficulty.

## Methods

Complete details of all UK Biobank procedures are available at www.ukbiobank.ac.uk.

### Ethics statement

This research was covered by the UK Biobank ethics agreement. Within England, UK Biobank has approval from the North West Multi-centre Research Ethics Committee (MREC). All participants provided written informed consent.

### Population and setting

Participants (n = 502,642; Table 1) volunteered between March 2007 and July 2010 following invitation letters to 9.2 million eligible UK residents [12]. Data were gathered at 22 assessment centres in England, Scotland and Wales. Participants attended a two-hour appointment during which they answered, via a touch-screen, questions about their life history, lifestyle and health, including their hearing. They also completed a hearing test and several tests of cognitive ability. Participants were free to opt out of the study at any time, either during the assessment or subsequently. Further details of UK Biobank recruitment, the DTT, and prevalence of hearing loss are presented elsewhere [31].

**Table 1:** Characteristics of UK Biobank participants.

Demographics	Female		Male		All	
	N (%)	mean±sd % +ve	N (%)	mean±sd % +ve	N (%)	mean±sd % +ve
Gender	273,448 (54%)	-	229,194 (46%)	-	502,642 (100%)	-
Age	273,448 (100%)	56.3±8.0 yrs	229,194 (100%)	56.7±8.2 yrs	502,642 (100%)	56.5±8.1 yrs
**Auditory tests/questions**						
SRT (better ear; dB)	87,650 (32%)	−7.4±1.6 dB	73,305 (32%)	−7.4±1.8 dB	160,955 (32%)	−7.4±1.7 dB
Hearing difficulty	258,576 (95%)	21%	219,120 (96%)	31%	477,696 (95%)	26%
Hearing in noise	266,966 (98%)	33%	224,032 (98%)	43%	490,998 (98%)	38%
Noisy workplace exposure	92,881 (34%)	11%	77,501 (34%)	37%	170,382 (34%)	23%
Loud music exposure	92,424 (34%)	9%	77,109 (34%)	17%	169,533 (34%)	12%
***Cognitive tests***						
Fluid Intelligence	90,202 (33%)	5.9±2.1 correct	75,306 (33%)	6.1±2.2 correct	165,508 (33%)	6.0±2.2 correct
Prospective Memory	93,382 (34%)	76%	78,219 (34%)	77%	171,601 (34%)	76%
Visual Memory	270,996 (99%)	4.7±3.7 pairs	227,054 (99%)	4.7±3.9 pairs	498,050 (99%)	4.7±3.8 pairs
Reaction Time	270,470 (99%)	567±117 ms	226,412 (99%)	551±116 ms	496,882 (99%)	560±117 ms
Digit Span	28,179 (10%)	6.4±1.8 digits	23,637 (10%)	6.6±1.9 digits	51,816 (10%)	6.5±1.8 digits

Number and performance of participants completing each auditory and cognitive measure. Percentages for each N other than gender are corrected for missing data. Mean ± sd: descriptive statistics for age, SRT, cognitive tests. % +ve: percent positive responses to each question. Cognitive test performance shows number of questions correctly answered (FI), % correct responses (PM), and number of incorrectly chosen pairs (VM).

### Hearing tests

All participants (Table 1) were asked “Do you have any difficulty with your hearing?” and “Do you find it difficult to follow a conversation if there is background noise (such as TV, radio, children playing)?” Possible answers were ‘Yes’; ‘No’; ‘Do not know’; ‘Prefer not to answer’. About one third of the participants were also asked “Have you ever worked in a noisy place where you had to shout to be heard?” and “Have you ever listened to music for more than 3 hours per week at a volume which you would need to shout to be heard or, if wearing headphones, someone else would need to shout for you to hear them?” Possible answers for these noise and music exposure questions were ‘Yes, for more than 5 years’; ‘Yes, for around 1–5 years’; ‘Yes, for less than a year’; ‘No’; ‘Do not know’; ‘Prefer not to answer’.

Guided by a video demonstration (see http://biobank.ctsu.ox.ac.uk/crystal/videos/hearing.swf), about one third of the participants (Table 1) completed a truncated DTT with fifteen (monosyllabic single digit) triplets (e.g. 5-0-8) presented separately to each ear via circumaural headphones (Sennheiser D25 [31]. A noise, shaped spectrally to the complete set of 9 digits, was played simultaneously. Both noise and (suprathreshold) speech levels were initially adjusted together to a comfortable level. The speech level was then fixed and noise level was varied adaptively after each triplet, dependent on the listener's correct touchscreen response to all three digits, to obtain criterion performance of 50% correct. The measure of hearing, the DTT SRT, was the mean signal-to-noise ratio from the last eight triplets. Testing of each ear took ∼4 minutes.

Participants were asked about several other aspects of hearing, including their use of hearing aids or cochlear implants. If they used hearing aids they were asked to remove them prior to completing the DTT. If they used cochlear implants they were asked not to attempt the test. Further studies using the UK Biobank resource and dealing in detail with associations and predictions between hearing and hearing aids [32], tinnitus [33], visual impairment and dual sensory problems [34], cigarette smoking and alcohol consumption [35], have or will be published elsewhere.

### Cognitive tests

Variable numbers of participants completed each test (Table 1), primarily due to the introduction of different tests at different stages during the UK Biobank project. The Fluid Intelligence test comprised thirteen questions designed to test logic and reasoning ability (e.g. If Truda's mother's brother is Tim's sister's father, what relation is Truda to Tim?; Fig. S3 in Results S1). The Prospective (long-term) Memory test presented the following instruction early in the cognitive test battery: “At the end of the games we will show you four coloured shapes and ask you to touch the Blue Square. However, to test your memory, we want you to actually touch the Orange Circle instead.” In two rounds, the Visual (short-term) Memory (‘Pairs matching’) test presented a matrix (1^st^ round: 2×3; 2^nd^ round: 3×4) of cards showing 3 and 6 pairs of shapes, respectively. The shapes were concealed and the participant was required to recall locations of matching shape pairs. Correct responses were confirmed. The Reaction Time test of processing speed sequentially presented twelve pairs of shapes. The participant pressed a button as quickly as possible if a pair of shapes matched. Finally, in the Digit Span (‘Numeric memory’) test of verbal working memory participants were initially presented on the screen with two digits and were required to key in the digits in reverse order. After each correct response the digit sequence was increased by one. The task ended after two incorrect responses. Raw score was the maximum number of correctly recalled and ordered digits. Performance on the Prospective Memory, Reaction Time and Digit Span tests additionally depended on executive function and all tests depended on alerting and orienting attention.

### Analysis and reporting

Analysis of self-report measures used binary logistic regression. Standardised scores, which each maximised the variability within the sampled population, were derived for each cognitive test using principal components analysis. A composite score was defined as a simple sum of the five standardised scores. Both individual and composite cognitive scores were standardised to have zero mean and unit standard deviation, such that higher values corresponded to higher ability. Scores were grouped into deciles for analysis. Regression modelling techniques accounting for sex, socio-economic deprivation (Townsend score, a proxy measure of socioeconomic status [31]), noise exposure (work and music), and interaction of socio-economic deprivation and noise with sex, were used to dissociate decline in hearing from decline in cognition with increasing age. Age at which full-time education was completed was not accounted for in these models but varied little between women (mean = 16.68, s.d. = 2.17) and men (mean = 16.76 years; s.d. = 2.52). Modelling that included cognitive scores was restricted to the 40,655 participants who completed all auditory and cognitive tests and questions. Data were assumed to be missing completely at random, as the primary reason for absent responses was the phased introduction of test components.

## Results

### Hearing

Age-related decline of hearing differs between test measures (Fig. 1). DTT SRT, averaged across participants, declined exponentially with age, starting from a low (sensitive) level in the 40 s (Fig. 1A). Both the absolute level and the rate of age-related decline of DTT SRT in men and women advanced in close parallel as individuals in the population became older, although SRT was slightly better in younger men than in younger women, and slightly better in older women than in older men. Tone sensitivity (indexed by the PTA, [9]) has also been shown in several studies across a 50 year time span to decline with age (Figs. 1B,C). However, in contrast to DTT SRT, tone sensitivity has previously been shown to decline rapidly through the 40 s, at least among men. Men had markedly poorer sensitivity than women at all ages >40, but women's sensitivity started to deteriorate in parallel with that of men when they reached their 60 s. A reduction in the rate of declining tone sensitivity with advancing age has been noted in some more recent studies (Fig. 1C [16], [18], [36]), but high frequency sensitivity remains much more susceptible to aging and clearly poorer in men (Fig. S1 in Results S1).

**Figure 2:**
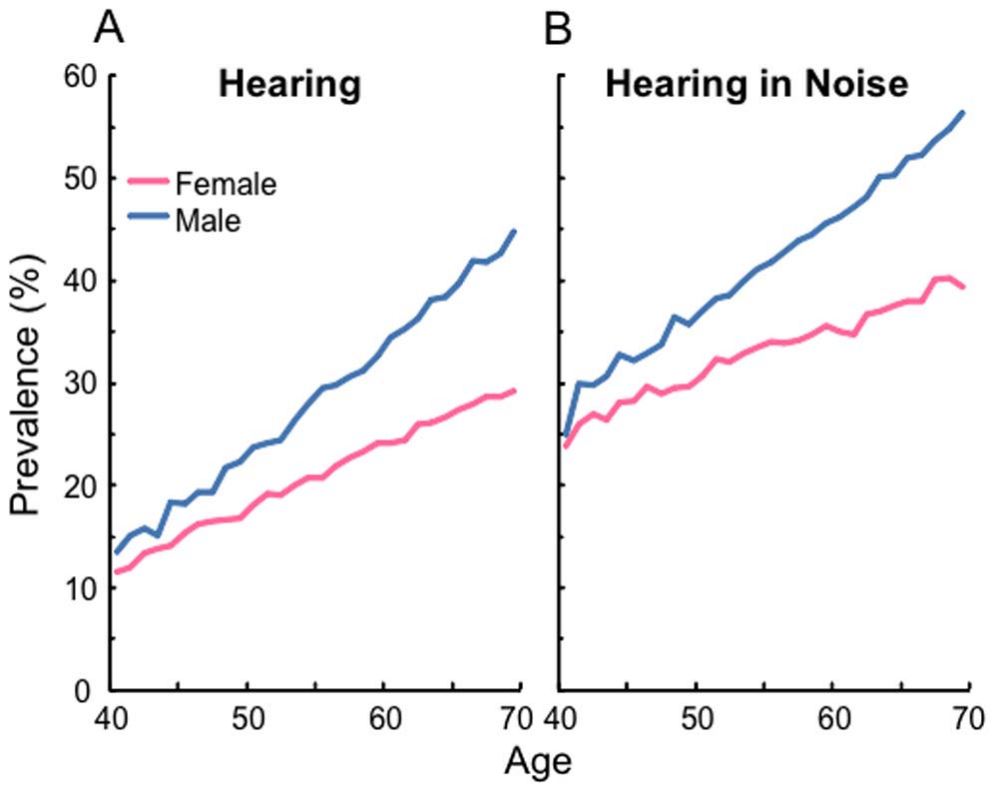
Men report greater difficulty hearing than women. Prevalence of self-report of (A) hearing difficulty and (B) difficulty hearing speech-in-noise in women and men from 40–70 y.o., corrected for socio-economic.

In UK Biobank, more men reported having problems with their hearing than did women (Fig. 2). Reported problems for both sexes grew linearly with age. By 69 y.o., men were reporting hearing difficulties, and difficulties in noise, at 40–60% higher prevalence than women. This increasing difference between men and women with age was not paralleled by objective measures of either speech or tone hearing (Fig. 1), nor by direct comparison between the prevalence of reported difficulty and DTT hearing (Fig. S2 in Results S1). Responses to questions about workplace noise (Table 2) showed the same relationship for women and men between increasing duration of noise exposure and the objective measure of decreased DTT SRT. Note, however, that the maximum effect size (mean 0.19 dB loss of SRT for >5 years exposure) was relatively small compared with age-related hearing loss (Fig. 1A; Table 3). No difference was found between the sexes for the effect of workplace noise on DTT SRT, and no significant effect of music exposure on DTT SRT was found. In contrast, both workplace noise and music exposure were significantly related to subjective reports of ‘difficulty hearing’ (Table 2) and ‘hearing in noise’ (Table S1 in Results S1). But while a far higher proportion of men than women reported exposure both to a noisy workplace and to loud music (Table 1), there was not a consistent dose-response difference between the sexes (Table 2). Men who experienced shorter noisy workplace exposure were less likely than women to report difficulty with their hearing, whereas men who experienced longer exposure were more likely than women to report difficulty. For music, there was no significant difference between the sexes.

**Table 2:** Noisy workplace and music exposure: objective and subjective effects on hearing.

Objective (SRT)	Female (dB)	p	Male (dB)	p	All (dB)	p	Factor (p)	Sex (p)
**Noisy workplace**	-	-	-	-	-	-	<0.001	0.572
<1year	-	-	-	-	0.05	0.10		
1–5 years	-	-	-	-	0.08	0.01		
>5 years	-	-	-	-	0.19	<0.001		
**Music exposure**	-	-	-	-	-	-	0.24	0.79
**Subjective (‘Difficulty’)**	Female (OR)	p	Male (OR)	p	All (OR)	p	Factor (p)	Sex (p)
**Noisy workplace**	-	-	-	-	-	-	<0.001	<0.001
<1year	1.72	<0.001	1.33	<0.001	-	-		
1–5 years	1.76	<0.001	1.84	<0.001	-	-		
>5 years	2.19	<0.001	2.89	<0.001	-	-		
**Music exposure**	-	-	-	-	-	-	<0.001	0.11
<1year	-	-	-	-	1.50	<0.001		
1–5 years	-	-	-	-	1.87	<0.001		
>5 years	-	-	-	-	1.98	<0.001		

Objective (SRT): General linear modelling of better ear speech reception threshold (dB) based on number of years workplace noise exposure relative to no exposure. Factor: Main effect for noise/music exposure. Sex: Interaction with gender. The model also includes terms for age, sex and cognition (Table 3).

Subjective (‘Difficulty hearing’): Logistic modelling of question association yielding odds ratios (OR) between no exposure and number of years indicated. Other details as for objective test. Similar results were obtained for ‘Difficulty hearing in noise’ (Table S1 in Results S1).

**Table 3:** Modelling speech-in-noise.

	Female		Male		All		Factor	Sex
	dB	p	dB	p	dB	p	p	p
**Gender** (Female)								
Male	-	-	-	-	−0.16	<0.001		
**Age** (40–44)	0	-	-	-	-	-	<0.001	<0.001
45–49	0.03	0.48	0.02	0.60	-	-		
50–54	0.12	0.001	0.16	<0.001	-	-		
55–59	0.31	<0.001	0.39	<0.001	-	-		
60–64	0.52	<0.001	0.66	<0.001	-	-		
65–69	0.75	<0.001	1.05	<0.001	-	-		
**Deprivation**	-	-	-	-	-	-	0.37	0.47
**Cognition** (1 = poor)	-	-	-	-	0	-	<0.001	0.39
2	-	-	-	-	−0.31	<0.001		
3	-	-	-	-	−0.43	<0.001		
4	-	-	-	-	−0.45	<0.001		
5	-	-	-	-	−0.51	<0.001		
6	-	-	-	-	−0.59	<0.001		
7	-	-	-	-	−0.59	<0.001		
8	-	-	-	-	−0.63	<0.001		
9	-	-	-	-	−0.63	<0.001		
10 = good	-	-	-	-	−0.70	<0.001		

General linear modelling of better ear speech reception threshold (dB) based on gender, age, socio-economic, and cognition. Gender, Age and Cognition reference levels were females, the youngest quintile, and the poorest performers, respectively. Factor: Main effect for Age/Deprivation/Cognition. Sex: Interaction with gender.

### Cognition

**Figure 3:**
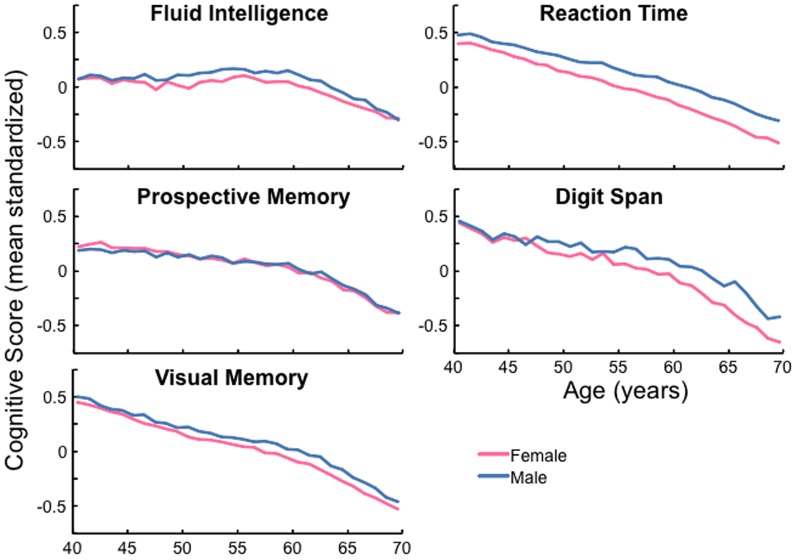
Cognitive performance declines with age. Cognitive performance of men and women in the UK Biobank study expressed as a mean standardized (z) score for ease of comparison between different tests.

Cognitive performance, like hearing, decreased with advancing age on all tests (Fig. 3, Table 3). For four tests, the pattern of decline was relatively simple and monotonic. However, Fluid Intelligence did not begin to decline until 60 y.o., then declined linearly for ages >60. Responses to individual Fluid Intelligence questions suggested this was due to a decline in both verbal and non-verbal performance from about this age (Fig. S3 in Results S1). Men performed better than women on all tests except prospective memory. The mean gap between the sexes increased with age on Reaction Time and Digit Span. Correlation between scores on individual tests varied from r = 0.19–0.38 (Table S2 in Results S1).

### Relation between hearing and cognition

**Figure 4:**
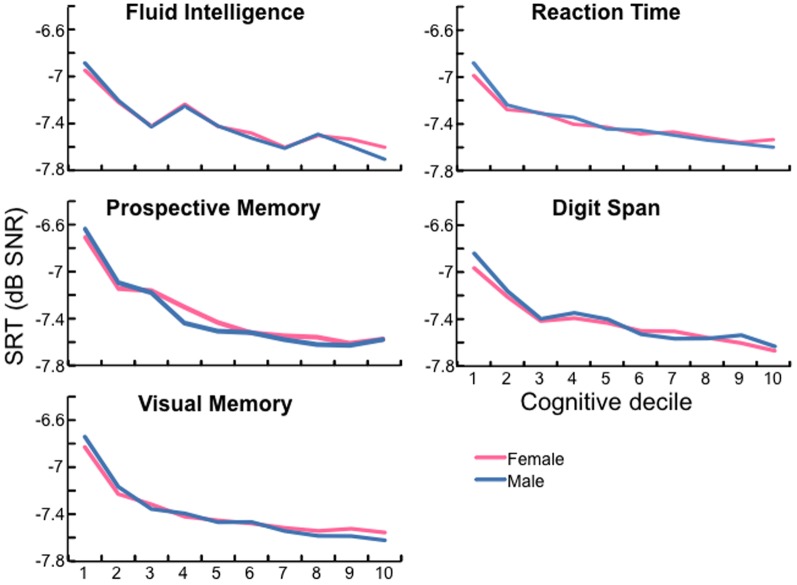
Better cognition is associated with better hearing. Relation between mean SRT and mean performance on each cognitive test (by decile of standardized score from 1 = low to 10 = high), all ages (40–69 y.o.) combined.

Better DTT SRT was associated with better cognitive ability. Fig. 4 shows cognitive ability on each test, plotted as deciles from 1 (lowest) to 10 (highest), as a function of DTT SRT. DTT SRT decreased by about 0.7 dB across the range of ability (i.e. from decile 1 to 10) on each cognitive test. This was similar in size to the increase in average DTT SRT across the 40–69 year age range (women: 0.75 dB, men: 1.05 dB; Table 3). DTT SRT differences were greatest at the lower end of cognitive ability. These results suggested that the ability to compensate for impaired DTT SRT depended strongly on cognitive performance, especially at the lower end of the spectrum. However, DTT SRT declined with age across the cognitive spectrum (Fig. S5 in Results S1).

## Discussion

### Hearing in quiet and in noise

We found that, on average, DTT SRT declines substantially during late middle age, confirming earlier studies [11], [20]. However, we also found little decline in DTT SRT until the 50 s, whereas declining PTA, based on the same frequency range used in the DTT, begins in the 30 s [14], [16], [18]. These differences may be due to the high ‘redundancy’ of the DTT speech signal relative to a single tone. Digits are a closed set of overlearned words. A listener can access many auditory tone channels, only some of which need be fully functional, to gain cues to digit identity. For example, many English digits can be distinguished using only vowels. Small changes in sensitivity to higher frequency tones in the 30 s and 40 s can therefore over-estimate real-life difficulty with speech, even when there is little or no contextual information, as in the DTT. At later ages, deficits in DTT SRT escalate rapidly and, by the early 60 s, are advancing relatively more rapidly than loss of tone sensitivity, presumably due to both more widespread loss of tone sensitivity and reduced auditory processing in the brain. Historically, tone sensitivity studies have also reported that men's hearing begins to deteriorate before that of women, particularly at high sound frequencies, and that the difference is maintained into old age. Some recent evidence [18] suggests that disparity may be declining, at least in the main speech range of hearing. Nevertheless, the UK Biobank data are notable in showing a close similarity between the DTT hearing of men and women across most of the age range studied, but with men showing a slightly greater decline in the late 60 s.

The different patterns of age-related hearing loss revealed by the two measures is evidence that DTT SRT utilizes different or additional mechanisms to those revealed by audiometry. One candidate mechanism may be an age-related loss of inner hair cell afferent synapses, as recently reported in mice [37]. In that example of supra-threshold hearing loss, mid-frequency pure tone thresholds measured by wave 1 of the auditory brainstem response were still normal in older (64–80 week old) mice when supra-threshold response amplitude was reduced relative to younger (4–16 week old) mice. The results suggest a loss of the number and synchrony of functional cochlear nerve fibres. In a human parallel of these findings, cochlear nerve neuron (spiral ganglion) cell bodies appear to be lost continually from a young age (<20 years [38]) when, again, auditory thresholds and otoacoustic emissions are normal, signifying intact hair cells. However, no differences in this process were seen between the sexes or across the length of the cochlea. Because aging men typically have poorer high frequency, but may not have poorer low frequency hearing than women (Fig. 1B; Fig. S1 in Results S1 [39]), we also suggest that the high redundancy of the speech signals enables men to identify the digits used in the current study based on the lower frequency cues (e.g. vowels) they can hear.

Subjective reports of hearing difficulty were predictable from DTT SRT, but did not match the pattern of decline in DTT SRT (or PTA [14]–[18]) with increasing age. A surprisingly high proportion of 40–50 y.o. people with normal DTT SRT indicated that they had difficulty hearing. That proportion rose with age and reduced DTT SRT, as expected, but the proportion of men reporting difficulty was markedly higher than that of women at nearly all ages. Two explanations for these phenomena are that the tests did not capture the handicap experienced, or that people misperceived their hearing to be worse than it really was. We think both these factors contributed to the results. Challenges to making realistic measures of speech hearing include the variety of situations encountered in everyday life and the variety of speech hearing to which individual brains are ‘tuned’. For example, we do most of our listening in reverberant rooms having a wide range of acoustic characteristics known to affect speech intelligibility [40]. Accents can be remarkably variable both between older, established communities and within modern, multi-ethnic urban centres, making calibration difficult, both for the audiologist and for the listener. The band-limited (<4 kHz) UK Biobank DTT probably captures some, but not all hearing loss that affects suprathreshold listening. Newly-developed, internet-deliverable SiN hearing tests that have shown greater sensitivity to high frequency hearing loss than the UK Biobank DTT [41], [42], may help further in meeting these challenges, and other SiN tests may capture additional relevant properties of the acoustic environment. For example, a modified version of the widely used QuickSIN [43] sentence-in-noise test incorporates separately measurable audiovisual, reverberation, and spatial cues, and a speeded speech condition [44] (see [45] for a review of SiN tests). Nevertheless, self-report remains an important ‘reality check’ on the usefulness of any measure of hearing.

This raises the second possibility, that people misperceive their degree of handicap. Everyone has difficulty hearing in some circumstances, and those circumstances are typically in a busy, noisy, reverberant room, such as a bar [40]. The UK Biobank data suggest this ‘baseline’ rate for perceived difficulty hearing in noise is around 25% for women and 35% for men (Fig. S2 in Results S1). There are many possible reasons why men may both have high estimates of their degree of handicap and have poorer tone sensitivity. One is that they may operate in noisier environments than women. In fact, more men than women reported spending time in noisy environments, both at work and at leisure, and men having longer exposure to noise also reported more difficulty hearing. Nevertheless, the DTT SRT of men did not differ significantly from exposure-matched women who made fewer reports of hearing difficulties, possibly because the men had an increased prevalence of auditory pathology that was not captured by the DTT SRT. Self-reported difficulty in relation to music exposure, which was significant, had quite high odds ratios, and did not differ between the sexes, was similarly not matched by any significant change in DTT SRT.

### Cognition and hearing

DTT SRT was found to decline with decreasing cognitive ability, consistent with previous reports using other SiN tests [46], [47]. If some aspect(s) of cognition (e.g. long-term memory [48]) was more important than others for speech-in-noise hearing and listening, that aspect should associate more strongly than others with DTT SRT. However, the decline was seen on each cognitive test used, to about the same extent, suggesting that some general cognitive factor may have been responsible. It has been suggested that reduced tone sensitivity is the ‘primary predictor’ of speech intelligibility with advancing age [49] and that age-related changes in cognitive function are mediated by age-related changes in ‘global sensory processing’ (hearing, vision, touch composite [47]). The current data suggest a much more prominent role for cognition in hearing, at least for the DTT. Other data from the NIH Toolbox, reported here for the first time, even suggest that poor cognitive function plays a role, albeit a relatively minor one, in reduced PTA (Fig. S4 in Results S1; [18]).

A critical question is whether decreasing tone sensitivity, decreasing cognitive function, both, or another factor (e.g. ‘common cause’ [50]) is primarily responsible for age-related decline of DTT SRT. Our analysis showed that, across the ages examined, better cognitive function was associated independently with a 0.7 dB reduction in DDT SRT. The effect of increasing age was additive to that effect, leading to a 0.7 (female) to 1.0 dB (male) increase in DDT SRT. In auditory terms, the decline in DTT SRT with age could be due to both peripheral and central factors. Peripheral hearing loss certainly contributes, since we know that tone sensitivity declines with age, even in the most cognitively able [50]. But declining central function may also contribute. For example, there is recent evidence that age-related decline in the maximum speed of auditory temporal processing includes a specifically central mechanism [51]. It is currently unknown whether this is operative within the central auditory system, which includes extensive sub-cortical networks, or within higher level processing areas that include those with multimodal cognitive functions. In either case, such a mechanism could have a marked effect on speech perception, since reduced temporal fine structure can reduce consonant discrimination [52]. Because of the high reliance of auditory processing on precise timing, auditory processing may prove a sensitive model for testing the ‘Processing speed theory’ of cognitive decline [53], according to which declining processing speed is the common cause of cognitive decline.

### Clinical significance

It is clear that cognitive factors play a major role in speech perception. Although even tone sensitivity is influenced by cognition (e.g. motivation, learning [54]), that influence is not strong, so there is an argument for the addition of tests of speech perception to routine audiometric assessment. Among the older population, differences in the cognitive abilities of the most and least able become more extreme [55] and reports of difficulties hearing speech in noisy environments become more common, as shown here and elsewhere [2]. SiN assessment may help identify those individuals who are likely to benefit from different interventions. Among middle-aged people, who do not typically seek assistance for their hearing loss [22], poorer SiN ability could be a first warning of a need for intervention. Unlike the audiogram, the DTT and some other SiN hearing tests can be delivered remotely for unsupervised, user-based testing via the internet, and are already being used for widespread hearing screening (e.g. visit actiononhearingloss.org.uk). A DTT (the Dutch Digits-in-Noise, DIN, test) has recently been suggested as a clinical diagnostic test [9]. Despite the close association with cognition demonstrated here, DTTs that use steady noise masking have been suggested to be less cognitively demanding than other SiN tests (e.g. using speech maskers [7]). However, this view has been questioned by DTT data [9] showing an extremely high correlation (r = 0.96, following level correction) with performance on Plomp and Mimpen [20] sentences. In contrast to other SiN tests, DTTs are also readily transferrable across cultural and even language groups (www.HearCom.eu [42], [10]). Together, these findings suggest the possibility of a DTT as an international standard for SiN testing.

## Supporting Information

Results S1 **This file contains Table S1, Table S2, and Figure S1–Figure S5.** Table S1, Noisy workplace and music exposure: Relation to ‘Hearing in noise’ question. Table S2, Pairwise Pearson correlation coefficients between cognitive test measures. Figure S1. Average (PTA) tone detection thresholds declined from 18–85 years (NIH Toolbox data [13]). Figure S2, Reported difficulty hearing increased with declining DTT hearing. Figure S3, Fluid intelligence varied with question, age and gender. Figure S4, PTA did not vary substantially with cognitive ability. Figure S5, Hearing declined with both cognitive ability and age.
(DOCX)
