# Supplementary material for: Relation between Speech-in-Noise Threshold, Hearing Loss and Cognition from 40–69 Years of Age
Source: PLoS One. 2014 Sep 17;9(9):e107720. doi: 10.1371/journal.pone.0107720 (PMC4168235; doi:10.1371/journal.pone.0107720)
Supplement: Results S1 — This file contains Table S1, Table S2, and Figure S1–Figure S5. Table S1, Noisy workplace and music exposure: Relation to ‘Hearing in noise’ question. Table S2, Pairwise Pearson correlation coefficients between cognitive test measures. Figure S1. Average (PTA) tone detection thresholds declined from 18–85 years (NIH Toolbox data [13]). Figure S2, Reported difficulty hearing increased with declining DTT hearing. Figure S3, Fluid intelligence varied with question, age and gender. Figure S4, PTA did not vary substantially with cognitive ability. Figure S5, Hearing declined with both cognitive ability and age. (DOCX) [file pone.0107720.s001.docx]

**Supporting Results S1**

**Supporting Table S1: Noisy workplace and music exposure: Relation to ‘Hearing in noise’ question**

Logistic modelling of question association yielding odds ratios (OR) between no exposure and number of years indicated. Other details in Table 2.

|  | **Female**  (OR) | p | **Male**  (OR) | p | **All**  (OR) | p | **Factor**  p | **Sex**  p |
| --- | --- | --- | --- | --- | --- | --- | --- | --- |
| **Noisy workplace** | 1 | - | 1 | - | - | - | <0.001 | 0.007 |
| < 1year | 1.832 | <0.001 | 1.294 | <0.001 | - | - |  |  |
| 1-5 years | 1.850 | <0.001 | 1.586 | <0.001 | - | - |  |  |
| >5 years | 2.168 | <0.001 | 2.225 | <0.001 | - | - |  |  |
| **Music exposure** | - | - | - | - | 1 | - | <0.001 | 0.172 |
| < 1year | - | - | - | - | 1.497 | <0.001 |  |  |
| 1-5 years | - | - | - | - | 1.769 | <0.001 |  |  |
| >5 years | - | - | - | - | 1.805 | <0.001 |  |  |

**Supporting Table S2: Pairwise Pearson correlation coefficients between cognitive test measures**

| **Test** | **Pairs Matching** | **Fluid Intelligence** | **Reaction Time** | **Numeric Memory** |
| --- | --- | --- | --- | --- |
| Prospective Memory | 0.306 | 0.325 | 0.244 | 0.382 |
| Pairs Matching | - | 0.266 | 0.265 | 0.344 |
| Fluid Intelligence | - | - | 0.193 | 0.334 |
| Reaction Time | - | - | - | 0.278 |
| Numeric Memory | - | - | - | - |

**Supporting Figure S1: Average (PTA) tone detection thresholds declined from 18-85 years** (NIH Toolbox data[[13](#_ENREF_13)])

A: PTA based on averaging lower tone frequencies (as in Figs. 1A,B), B: PTA based on averaging higher tone frequencies.

**Supporting Figure S2: Reported difficulty hearing increased with declining DTT hearing**

Proportion of participants with hearing difficulties having SRT within each 0.5 dB bin from -10 to -2.5 dB, all ages combined. Too few participants had SRTs outside this range to permit interpretation. Men reported more difficulty hearing, and hearing in noise. However, the increasing prevalence of reported hearing difficulty with declining DTT hearing (less negative SRT) occurred in parallel for men and women.

**Supporting Figure S3: Fluid intelligence varied with question, age and gender**

Responses of women and men to representative individual questions in the Fluid Intelligence test. In addition to the answer options shown, ‘Do not know’ and ‘Prefer not to answer’ were available for all questions. Thirteen questions were asked with a mean of 8.0 (40 y.o.) – 7.2 (69 y.o.) answered within 2 minutes. Percentage correct responses here for Q3 – Q9 were of the total number of participants who answered any questions (text Table 1). Thus, for Q5, 48% of those attempting the question (n = 160,086) answered correctly, while for Q9, 55% of those attempting the question (n = 47,076) answered correctly.

**Supporting Figure S4: PTA did not vary substantially with cognitive ability**

NIH Toolbox data[[13](#_ENREF_13)] showing the relationship, across ages 18-85 years, between audibility (PTA at 0.5, 1, 2 and 4 kHz) and cognitive composite score (standardized by quintile, 1 = low, 5 = high). Women had more sensitive audibility across the range and higher ability individuals of both sexes had better thresholds. Note, however, the relatively small and linear change in PTA compared with SRT (c.f. Figs 1B,C, 4).

**Supporting Figure S5: Hearing declined with both cognitive ability and age**

Mean SRT in each decade as a function of mean standard score on all cognitive tests (1 = low, 10 = high).
